# Phase I trial of TRC102 (methoxyamine HCl) in combination with temozolomide in patients with relapsed solid tumors and lymphomas

**DOI:** 10.18632/oncotarget.27784

**Published:** 2020-11-03

**Authors:** Geraldine O'Sullivan Coyne, Shivaani Kummar, Robert S. Meehan, Khanh Do, Jerry M. Collins, Larry Anderson, Kazusa Ishii, Naoko Takebe, Jennifer Zlott, Lamin Juwara, Richard Piekarz, Howard Streicher, Elad Sharon, Larry Rubinstein, Andrea Regier Voth, Jay Lozier, Angie B. Dull, Deborah Wilsker, Toshinori Hinoue, Peter W. Laird, Katherine V. Ferry-Galow, Robert J. Kinders, Ralph E. Parchment, James H. Doroshow, Alice P. Chen

**Affiliations:** ^1^Early Clinical Trials Development Program, Division of Cancer Treatment and Diagnosis, National Cancer Institute, National Institutes of Health, Bethesda, MD, USA; ^2^Division of Cancer Treatment and Diagnosis, National Cancer Institute, Rockville, MD, USA; ^3^Hematology Branch, National Heart, Lung, and Blood Institute, National Institutes of Health, Bethesda, MD, USA; ^4^Clinical Monitoring Research Program Directorate, Frederick National Laboratory for Cancer Research, Frederick, MD, USA; ^5^Cancer Therapy Evaluation Program, Division of Cancer Treatment and Diagnosis, National Cancer Institute, National Institutes of Health, Bethesda, MD, USA; ^6^Biometric Research Program, Division of Cancer Treatment and Diagnosis, National Cancer Institute, Bethesda, MD, USA; ^7^Applied/Developmental Research Directorate, Frederick National Laboratory for Cancer Research, Frederick, MD, USA; ^8^Pediatric Oncology Branch, National Cancer Institute, National Institutes of Health, Bethesda, MD, USA; ^9^Clinical Pharmacodynamic Biomarkers Program, Applied/Developmental Research Directorate, Frederick National Laboratory for Cancer Research, Frederick, MD, USA; ^10^Van Andel Institute, Center for Epigenetics, Grand Rapids, MI, USA; ^11^Current address: Knight Cancer Institute, Oregon Health Sciences University, Portland, OR, USA; ^12^Division of Cancer Treatment and Diagnosis, and Center for Cancer Research, National Cancer Institute, National Institutes of Health, Bethesda, MD, USA

**Keywords:** DNA damage repair, base excision repair, MGMT, rational combination therapy, molecular pharmacodynamics

## Abstract

Background: TRC102 inhibits base excision repair by binding abasic sites and preventing AP endonuclease processing; it potentiates the activity of alkylating agents, including temozolomide, in murine models. In published xenograft studies, TRC102 enhanced the antitumor effect of temozolomide regardless of cell line genetic characteristics, e.g., O6-methylguanine DNA methyltransferase (MGMT), mismatch repair (MMR), or p53 status.

Materials and Methods: We conducted a phase 1 trial of TRC102 with temozolomide given orally on days 1-5 of 28-day cycles in adult patients with refractory solid tumors that had progressed on standard therapy. Tumor induction of nuclear biomarkers of DNA damage response (DDR) γH2AX, pNBs1, and Rad51 was assessed in the context of MGMT and MMR protein expression for expansion cohort patients.

Results: Fifty-two patients were enrolled (37 escalation, 15 expansion) with 51 evaluable for response. The recommended phase 2 dose was 125 mg TRC102, 150 mg/m^2^ temozolomide QDx5. Common adverse events (grade 3/4) included anemia (19%), lymphopenia (12%), and neutropenia (10%). Four patients achieved partial responses (1 non-small cell lung cancer, 2 granulosa cell ovarian cancer, and 1 colon cancer) and 13 patients had a best response of stable disease. Retrospective analysis of 15 expansion cohort patients did not demonstrate a correlation between low tumor MGMT expression and patient response, but treatment induced nuclear Rad51 responses in 6 of 12 patients.

Conclusions: The combination of TRC 102 with temozolomide is active, with 4 of 51 patients experiencing a partial response and 13 of 51 experiencing stable disease, and the side effect profile is manageable.

## INTRODUCTION

Among the various mechanisms by which resistance to chemotherapy can develop, aberrations in the BER pathway have been reported to play a major role in promoting resistance to alkylating and antimetabolite chemotherapy [1]. BER is triggered by the DNA glycosylase enzymes that recognize and remove damaged DNA bases, creating an AP site that APE processes further by nicking the adjacent phosphodiester DNA backbone [2]. TRC102 (methoxyamine hydrochloride) is a novel small molecule amine that can interrupt the BER process by covalently binding to the reactive aldehyde created in AP sites, which blocks the catalytic activity of APE and inhibits completion of BER [3].

The ability of TRC102 to potentiate the cytotoxicity of chemotherapy was initially demonstrated using the alkylating agent temozolomide (TMZ) [4, 5]. TMZ alkylation produces several adducts, including N^7^-methylguanine and N^3^-methyladenine, which constitute ~80% of the adducts produced and are efficiently removed by BER pathway glycosylases, providing the rationale for the combination of these agents (Supplementary Figure 1) [3, 6]. However, TMZ also alkylates guanine at the O^6^ position, producing the well-known O^6^-methylguanine (O^6^-MG) lesion, which incorrectly base pairs with thymine [7]. Recognition of this error by the MGMT enzyme allows for the direct removal of the methyl/alkyl group from the O^6^ position by the same protein and restores guanine to its normal form, making MGMT critical to single-agent TMZ resistance [8]. The accumulation of O^6^-MG:T mismatches due to absent or low MGMT expression causes a futile cycle of repeated repair attempts by the MMR machinery on O^6^-MG:T base pairs that eventually creates secondary DNA strand breaks leading to cellular apoptosis [9, 10]. Therefore, the combination of low tumor MGMT expression and a functional MMR pathway is often considered necessary for TMZ activity, tumor characteristics that only a subset of patients demonstrate.

In published studies, TRC102 was found to potentiate the antitumor effect of TMZ and carmustine in several murine xenograft models of colon cancer, regardless of cell line genetics, including the status of MGMT, MMR, or p53, potentially providing a route to MGMT status-independent TMZ activity [5]. On the basis of this data, we conducted a phase 1 trial of oral TRC102 in combination with oral TMZ in patients with refractory solid tumors to determine the regimen’s safety, MTD, RP2D, PK, molecular pharmacodynamics, and antitumor activity.

## RESULTS

### Patient population and disposition

A total of 52 patients were enrolled in the phase 1 portion of this trial between July 2013 and November 2016, including a 15-patient expansion cohort (Table 1). Median age was 59 years (range, 38–83 years) and all patients had been previously treated, with a median of 4 prior lines of therapy (range 1–12). One patient did not begin treatment, leaving 51 patients evaluable for response. Five patients stopped treatment by choice, one patient was removed at the discretion of the PI, and one patient came off-study due to intercurrent illness (a stroke, determined to be unrelated to study treatment). One patient died on study, and three patients died during the 30-day follow-up period, all due to disease progression.

**Table 1:** Patient population

**Patient Characteristics**	
Number of patients enrolled/evaluable	52/51
Median age, years (range)	59 (38–83)
ECOG Performance status	
0	5
1	47
Sex	
Male	27 (52%)
Female	25 (48%)
Diagnosis	
Colorectal cancer	16
Ovarian cancer	6
Granulosa cell of the ovary	2
Mesothelioma	4
Breast cancer	3
Cholangiocarcinoma	3
Miscellaneous solid tumors	20
Median number of prior therapies (range)	4 (1–12)
Patients with ≥ 1 prior alkylating agent(s)	42

**Figure 1:**
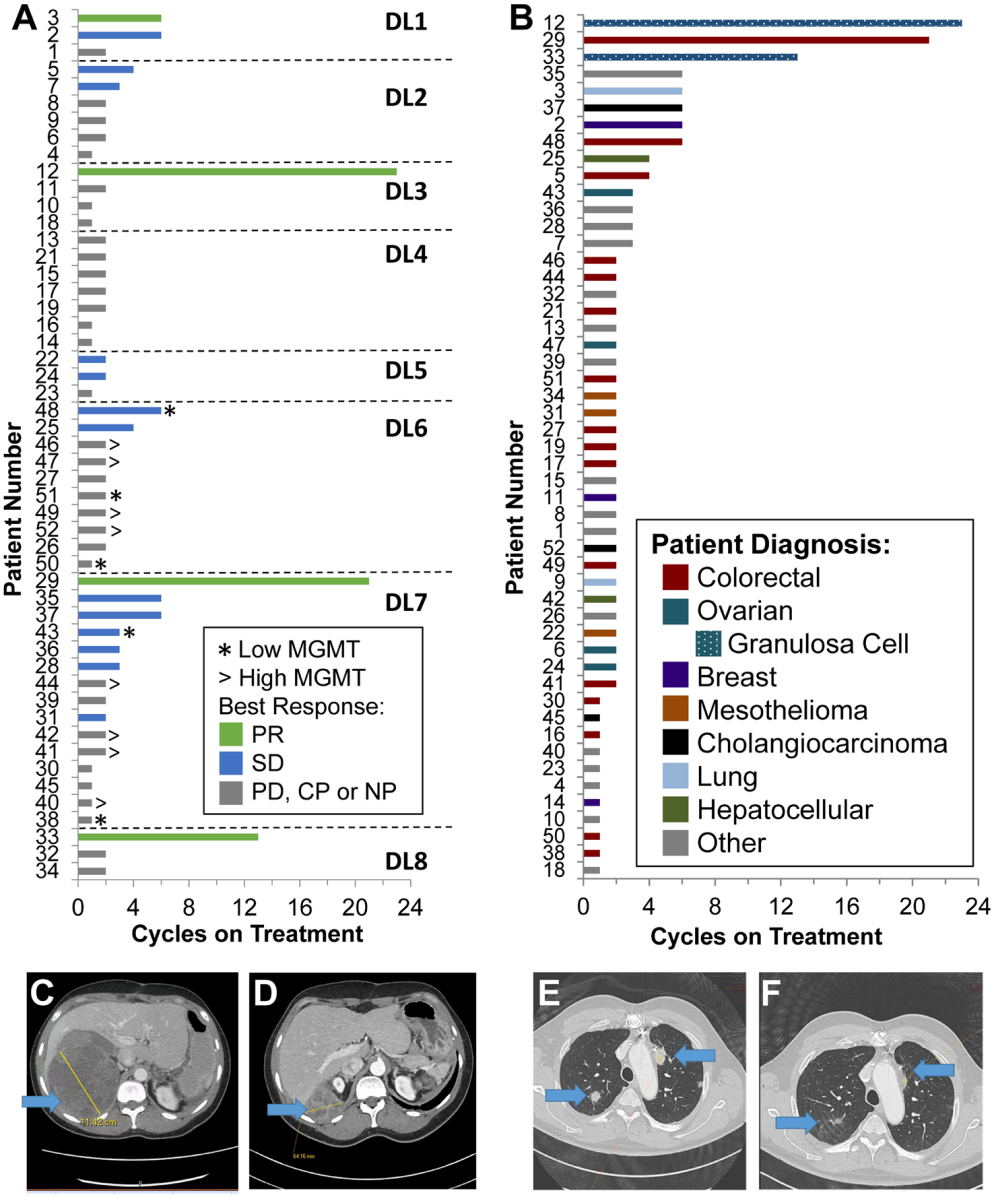
Patient outcomes. (**A**) All patients eligible for evaluation of response sorted by dose level and time on treatment with best response shown (PR, partial response; SD, stable disease; PD, progressive disease; CP, clinical progression; NP, not per protocol, i.e., no scan), as well as tumor MGMT expression level for those patients in which a tumor biopsy was available for assessment (using IHC with 30% positive nuclear staining defining the cutoff between high and low). (**B**) All patients eligible for evaluation of response sorted by time on treatment and colored by diagnosis. Note that patients with granulosa cell ovarian cancer are included as a subset of patients with ovarian cancers. (**C**) Baseline target extra (hepatic) capsular lesion in a patient with granulosa cell ovarian cancer (patient 12). (**D**) Restaging scans from patient 12 after 14 cycles of therapy. (**E**) Baseline target lung parenchymal lesions in a patient with colorectal carcinoma (patient 29). (**F**) Restaging scans from patient 29 after 2 cycles of therapy.

The mean time on study was 3.3 cycles, with a range of 1 to 23 cycles (Figure 1A and 1B). While 15 patients had clinical progression prior to the first restaging and 21 patients were found to have radiologic or clinical progression at the first restaging, four patients had confirmed PRs (one patient with NSCLC, one patient with colon cancer, and two patients with granulosa cell ovarian cancer), and eleven patients had a best response of stable disease. Both patients with non-epithelial ovarian cancer experiencing confirmed PRs were post-menopausal patients diagnosed with granulosa cell tumors of the ovary, adult subtype. The first was a 50-year-old patient diagnosed with stage IC disease who had undergone multiple laparotomies together with platinum-based therapy but eventually developed widespread peritoneal disease. She had progressed on two prior phase I trials before enrolling on this study. She was treated at DL3 (50 mg TRC102 and 150 mg/m^2^ TMZ) and experienced a PR at her first restaging and remained on study for 23 cycles (Figure 1C and 1D). The second was a 51-year-old patient who had received four prior lines of therapy for widely metastatic disease. She was treated at DL8 (150 mg TRC102 and 200 mg/m^2^ TMZ) and experienced a DLT with grade 3 anemia during cycle 1. Her laboratory evaluations during this event did not reveal hyperbilirubinemia or reduced haptoglobin levels. She was dose reduced to DL7 (150 mg TRC102 and 150 mg/m^2^ TMZ), experienced a PR at her first restaging, and remained on study for 13 cycles.

One 64-year-old patient with Stage IV squamous cell carcinoma of the lung, treated with definitive chemoradiotherapy in the adjuvant setting and 2 lines of platinum-based therapy in the metastatic setting, had a confirmed PR while on DL1 (25 mg TRC102 and 125 mg/m^2^ TMZ). He had a greater than 50% reduction in his disease at first restaging and remained on study for 6 cycles. A 68-year-old patient with *KRAS*-mutated advanced colon cancer, who received two prior lines of therapy in the palliative setting, was initially treated at DL7. He experienced a PR at his first restaging (Figure 1E and 1F), followed by continued reduction of disease with a near CR; he chose to come off study after 21 cycles of therapy. He was dose-reduced to DL6 (125 mg TRC102 and 150 mg/m^2^ TMZ) due to grade 3 nausea at cycle 6, and then subsequently to 4 days of therapy, with no progression. At last contact, he had been followed off therapy for 2 years without evidence of progression.

### Toxicity

This treatment regimen was very well tolerated. Most adverse events were grade 1 and 2. The most common grade 3 or higher adverse events at least possibly related to study drugs were anemia (10 patients) and lymphopenia (6 patients). Other common adverse events were neutropenia (5 patients), thrombocytopenia (3 patients), and leucopenia (3 patients) (Table 2). DL7 was established as the MTD after two DLTs (grade 3 hemolysis and abdominal pain) occurred on DL8; however, 5 of 8 patients in the DL7 expansion cohort had grade 3 anemia in the first cycle and required transfusion; therefore, the remaining expansion cohort patients received DL6. None of the latter patients required transfusions, and DL6 was ultimately determined to be the RP2D for this combination.

**Table 2:** Adverse events occurring in >3% of patients

Adverse Event	Number of Patients	
	Grade 3	Grade 4
Anemia	10	
Aspartate aminotransferase increased	2	
Fatigue	1	
Hemolysis	2	
Hypophosphatemia	1	
Lymphocyte count decreased	5	1
Nausea	1	
Neutrophil count decreased	2	3
Platelet count decreased		3
Vomiting	1	
White blood cell decreased	3	

Worst grade (≥ 2) that is at least possibly related to study drugs is shown for each patient (*N* = 52 total patients).

Anemia was an expected hematological toxicity for TRC102 based on pre-clinical studies and a prior phase I/II study of oral TRC102 with pemetrexed. In pre-clinical studies, anemia due to extravascular hemolysis in the spleen was observed, as indicated by decreased plasma haptoglobin, and serum levels of bilirubin together with a hypercellular marrow and reticulocytosis [11]. Given this background and the observation of grade 3 hemolysis at DL7, we incorporated a comprehensive hemolysis workup together with specialty erythrocyte testing in the expansion cohort. A total of 7 patients underwent this workup, and 4 patients had a reduction in haptoglobin. No alterations in RBC enzymes, methemoglobin, G6PD, or osmotic frailty were detected.

### Pharmacokinetics

**Figure 2:**
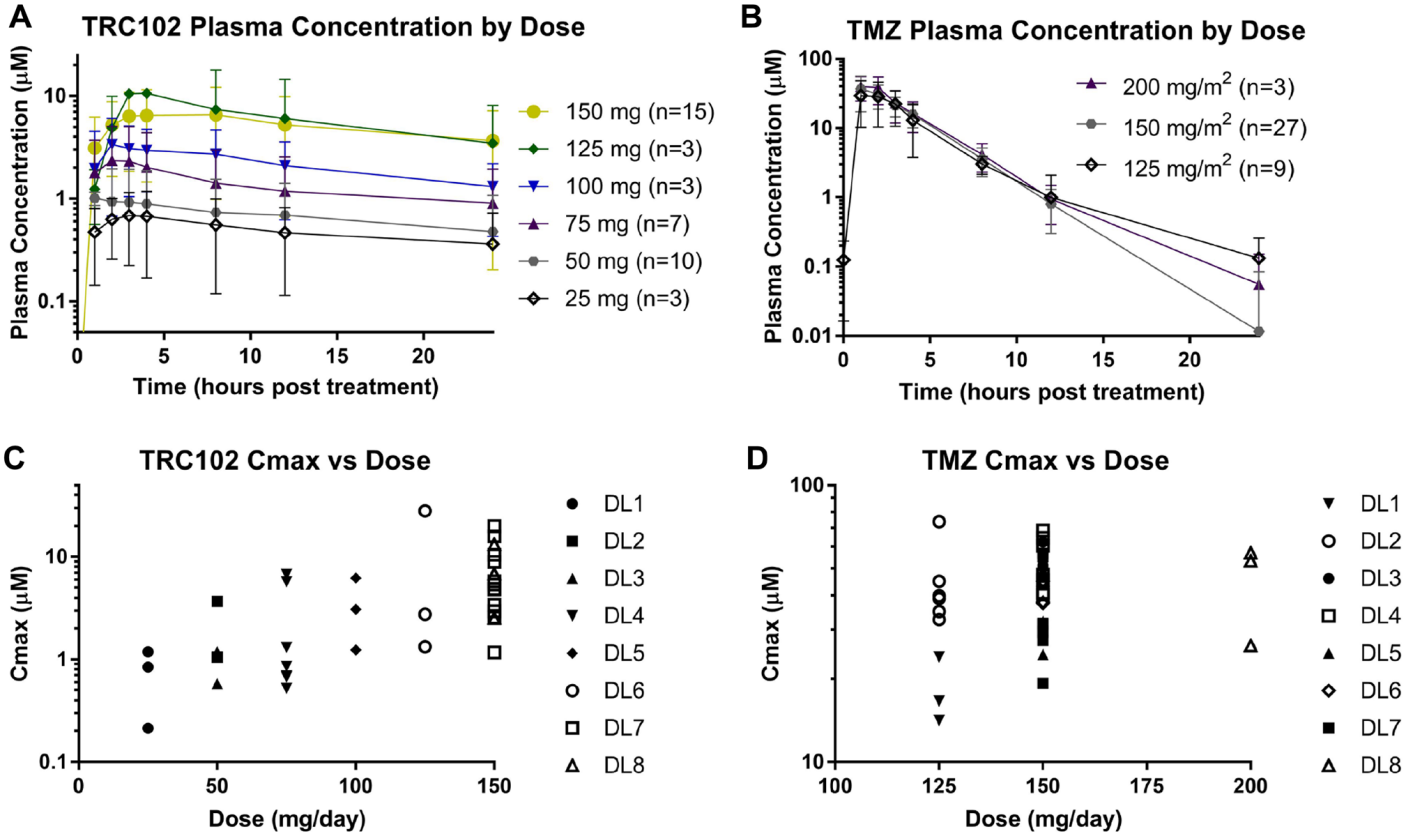
Pharmacokinetic analysis. Semi-log plots showing the average plasma concentration over time from patients receiving their first dose of (**A**) TRC102 and (**B**) TMZ grouped by dose level (error bars indicate standard deviation). Semi-log plots of the maximal plasma concentrations (C_max_) for each patient after receiving their first dose of (**C**) TRC102 and (**D**) TMZ.

Analysis of the PK data indicate that all dose levels of TRC102 reached C_max_ > 50 ng/mL, which was required for *in vivo* activity in preclinical models. Further, the PK profile of both TRC102 and TMZ observed here appear to be similar to prior studies [11–13] with a half-life of 26 hours for TRC102 and 2 hours for TMZ; however, in the absence of additional single-agent groups in the current study, it is not feasible to definitively state that there were no PK-based interactions between these agents (Figure 2).

### MGMT protein expression in tumor cells

**Figure 3:**
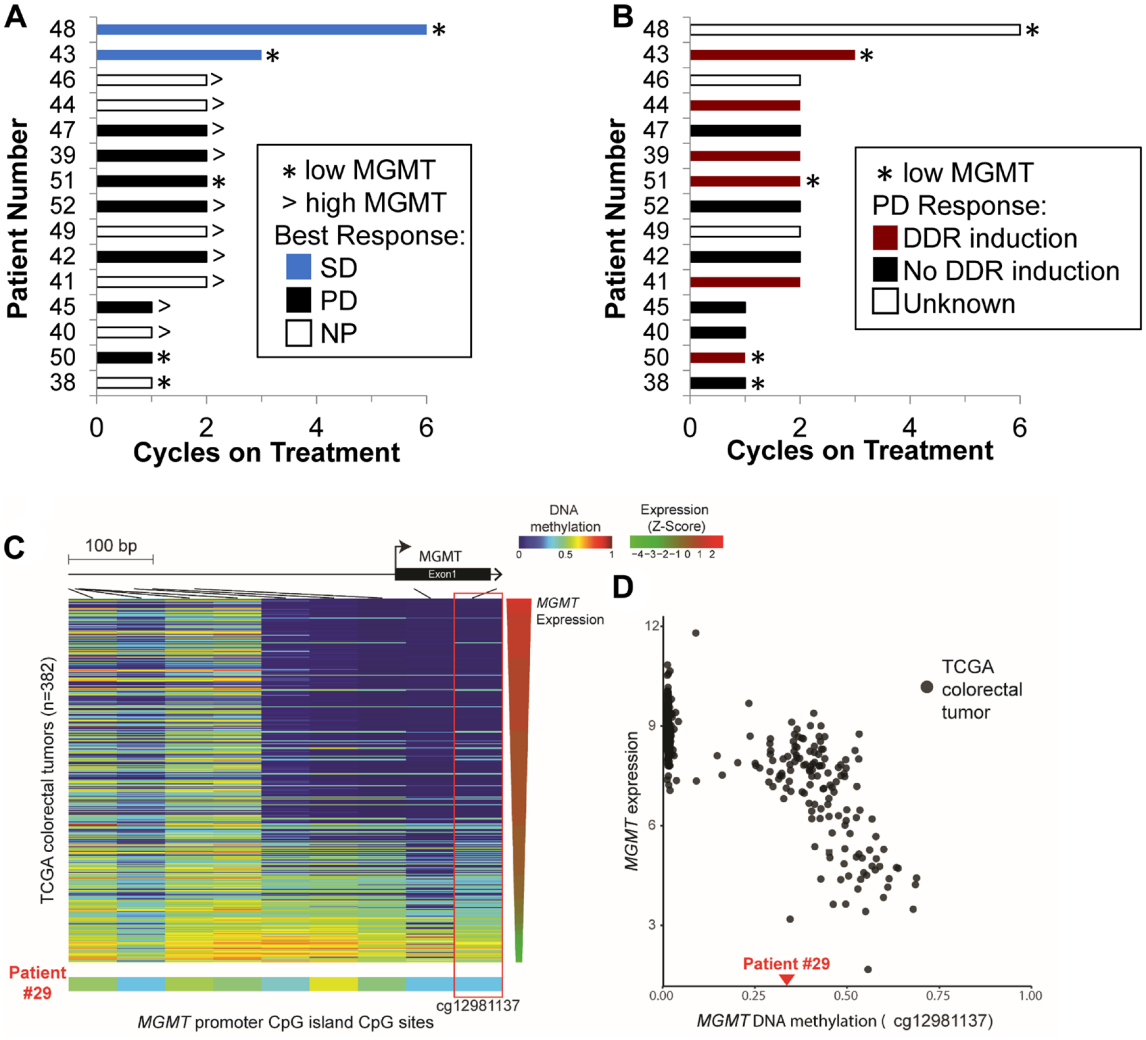
MGMT status and pharmacodynamic response. Expansion cohort patients sorted by time on treatment with MGMT status and either (**A**) best response or (**B**) induction of one or more nuclear DNA damage repair (DDR) biomarkers (γH2AX, pNbs1, or Rad51) shown. (**C**) Heatmap displaying DNA methylation patterns at *MGMT* promoter CpG islands in TCGA colorectal tumors (top) and tumor tissue from patient 29 (bottom). The DNA methylation β values are represented by using a color scale from dark blue (low DNA methylation) to red (high DNA methylation). TCGA tumors are arranged from top to bottom in order of decreasing *MGMT* expression level represented as Z-Score, with green indicating low expression and red high expression. DNA methylation is evident in the patient 29 tumor across all CpG sites examined. The red box highlights probe cg12981137, which was selected by Knijnenburg *et al.* in a previous TCGA study as the optimal probe for *MGMT* status identification [43]. (**D**) Scatter plot exhibiting an inverse relationship between DNA methylation (cg12981137) and *MGMT* expression [log_2_(RSEM+1)] in TCGA colorectal tumors. The DNA methylation level of patient 29 (β = 0.34) is indicated by the red triangle on the horizontal axis. The DNA methylation level at this diagnostic CpG of patient 29 falls within the range of TCGA colorectal tumors demonstrating *MGMT* epigenetic silencing.

Tumor biopsies were collected from 15 patients in the expansion cohort (DL6 and DL7). All baseline biopsies were assessed for MGMT protein expression by IHC (Figure 3A) and no statistically significant relationship was established between MGMT expression and patient outcome; the most suggestive finding was that the relationship between low MGMT expression and stable disease approached significance by 2-sided Fisher’s Exact Test (*p=* 0.10). The lack of statistical significance may have been due to the lack of response of patients in the expansion cohort (only 2 patients achieved a best response of stable disease and none had partial responses) and the fact that 3 patients in this group chose to come off study in the first 2 cycles, including 1 patient with low MGMT expression (Table 3 and Figure 3A).

**Table 3:** PD summary

Patient Number^†^and Best Response	Patient Diagnosis, Biopsy Site, and Timing of On-treatment Biopsy	MGMT^‡^and MMR^§^Status (by IHC)	γH2AX (mean %NAP)	pNbs1 (mean %NAP)	Rad51 (% cells ≥ 5 foci)
**38**	Dx: Colon	MGMT Low	Pre: 7.7	Pre: 0.75	Pre: 0.4
**NP**	Bx: Liver (C1D4)	MMR Proficient	On-Tx: 3.9	On-Tx: 0.85	On-Tx: 0.7
**39**	Dx: Cervical	MGMT High	Pre: 1.1	Pre: 1.2	Pre: 1.7
**PD**	Bx: Liver (C1D4)	NA^§^	On-Tx: 0.7	On-Tx: 0.6	On-Tx: 5.8^*^
**40**	Dx: Bladder cancer	MGMT High	Pre: 3.6	Pre: 0.9	Pre: 1.8
**NP**	Bx: Right Inguinal Node (C1D4)	NA^§^	On-Tx: 1.3	On-Tx: 0.1	On-Tx: 0.1
**41**	Dx: Colon	MGMT High	Pre: 4.7	Pre: 1.7	Pre: 2.0
**NP**	Bx: Liver (C1D4)	NA^§^	On-Tx: 5.2	On-Tx: 1.8	On-Tx: 10.6^*^
**42**	Dx: Hepatocellular	MGMT High	Pre: 0.7	Pre: 0.5	Pre: 0.1
**PD**	Bx: Liver (C1D5)	NA^§^	On-Tx: 0.7	On-Tx: 0.5	On-Tx: 0.1
**43**	Dx: Ovarian	MGMT Low	Pre: 1.2	Pre: 1.2	Pre: 0.5
**SD (3 cycles)**	Bx: Liver (C1D4)	MMR Proficient	On-Tx: 1.1	On-Tx: 4.6	On-Tx: 6.0^*^
**44**	Dx: Colon	MGMT High	Pre: 0.4	Pre: 0.9	Pre: 0.5
**CP**	Bx: Liver (C1D5)	NA^§^	On-Tx: 14.1^*^	On-Tx: 3.5	On-Tx: 7.1^*^
**45**	Dx: Cholangiocarcinoma	MGMT High	Pre: 0.5	Pre: 0.2	Pre: 0.2
**PD**	Bx: Liver (C1D5)	NA^§^	On-Tx: 0.6	On-Tx: 2.4	On-Tx: 1.8
**46**	Dx: Colon	MGMT High	NA^†^	NA^†^	NA^†^
**NP**	Bx: Liver (C1D4)	NA^§^			
**47**	Dx: Ovarian	MGMT High	Pre: 0.2	Pre: 7.1	Pre: 1.2
**PD**	Bx: Lower pelvic mass right side (21 hours after C1D5 tx)	NA^§^	On-Tx: 0.4	On-Tx: 3.6	On-Tx: 1.9
**48**	Dx: Rectal	MGMT Low	NA^†^	NA^†^	NA^†^
**SD (6 cycles)**	Bx: Retroperitoneal lymph node (21 hours after C1D5 tx)	MMR Proficient			
**49**	Dx: Colon	MGMT High	NA^†^	NA^†^	NA^†^
**NP**	Bx: Liver (C1D5)	NA^§^			
**50**	Dx: Colon	MGMT Low	Pre: 0.8	Pre: 4.9	Pre: 5.6
**PD**	Bx: Liver (C1D4)	MMR Proficient	On-Tx: 2.9	On-Tx: 10.2^*^	On-Tx: 16.0^*^
**51**	Dx: Colon	MGMT Low	Pre: 3.3	Pre: 1.3	Pre: 0.5
**PD**	Bx: Liver (C1D4)	MMR Proficient	On-Tx: 1.8	On-Tx: 11.3^*^	On-Tx: 17.0^*^
**52**	Dx: Liver adenocarcinoma	MGMT High	Pre: 0.01	Pre: 4.2	Pre: 9.2
**PD**	Bx: Liver (23 hours after C1D5 tx)	NA^§^	On-Tx: 0.01	On-Tx: 5.3	On-Tx: 12.4

^**†**^All patients from whom baseline tumor biopsies adequate for analysis were collected are included here, but for quality control purposes, DDR biomarkers were only analyzed in matched biopsy pairs deemed by pathologist review of H&E stained sections to contain adequate tumor content and quality. ^‡^Baseline biopsies were assessed by IHC for MGMT expression levels with a cutoff of 30% nuclear staining. ^§^Baseline biopsies with low MGMT protein levels were further assessed for the expression of the MMR proteins MLH1, MSH2, MSH6, and PMS2 by IHC; tumors expressing all 4 proteins were considered MMR proficient. Baseline biopsies with high MGMT protein levels were not assessed for MMR proteins. ^*^Significant elevations of biomarkers on-treatment (defined as ≥ 5% of tumor cells exhibiting ≥5 Rad51 nuclear foci or ≥4% of tumor nuclear area positive for γH2AX or pNbs1 staining [14]) are marked with asterisks. Abbreviations: Bx, biopsy site; CP, clinical progression; Dx, diagnosis; NP, not per protocol (i.e., no measurement of disease response available because patient refused further treatment); PD, progressive disease; Pre, biopsy taken before initiation of treatment; On-Tx, biopsy collected on cycle 1, day 4 or 5, 3–4 hours after treatment unless otherwise specified; SD, stable disease; Tx, treatment; %NAP, percent tumor nuclear area positive for the given biomarker.

MGMT promoter methylation status was also available for one escalation phase patient. The patient with colon cancer who achieved a confirmed PR (DL7) enrolled in the NCI Exceptional Responder pilot study (NCT02243592), which retrospectively aims to elucidate possible molecular mechanisms of response to therapy (manuscript in preparation). He provided informed consent for sharing of assay results across trials under the Longitudinal Sample Collection and Tracking protocol at the NCI, allowing us to document epigenetic silencing of *MGMT* in his archival tumor tissue (Figure 3C and 3D).

### MMR protein expression in tumor cells with low MGMT

Follow-up assessment of baseline biopsies from all expansion cohort patients with low tumor MGMT expression revealed robust expression of MLH1, MSH2, MSH6, and PMS2 proteins in 5 of 5 cases (data not shown), likely indicating a functional MMR response; however, the assay results were not informative regarding the patient response to treatment on this study.

### Pharmacodynamics

In the expansion cohort, evaluable pairs of pre- and on-treatment biopsies suitable for analysis of DDR biomarkers were obtained in cycle 1 from 12 of 15 patients. After 4–5 days of combination drug treatment, nuclear Rad51, a homologous recombination biomarker, was significantly elevated in biopsies from 6 of the 12 patients. Elevated Rad51 levels were defined by the observation that at least 5% of tumor cells assessed demonstrated ≥ 5 Rad51 nuclear foci (Table 3) [14]. Among these 6 cases, 3 also exhibited elevated nuclear γH2AX or pNbs1 (defined as ≥ 4% of tumor nuclear area positive for γH2AX or pNbs1 staining) [14]. Of note, 4 of 5 patients in the expansion cohort with colorectal cancer exhibited a nuclear DDR biomarker response of some type.

For the 5 patients with low tumor MGMT expression, 3 displayed a nuclear DDR biomarker response to treatment, whereas 1 did not and 1 was not assessable due to insufficient tumor tissue in the on-treatment biopsy (Figure 3B). The combination of low tumor MGMT expression and nuclear DDR biomarker induction did not correlate with clinical response in this small test group—2 of 3 MGMT^low^/DDR^+^ patients progressed within the first 2 cycles of treatment (patients 50 and 51, both patients with colon cancer).

## DISCUSSION

TMZ has been in use for 30 years but is only FDA-approved for the treatment of GBM and melanoma. This is due, in part, to the specialized testing needed to identify patients with MGMT-deficient tumors. Although MGMT-deficiency is particularly common in GBM, other tumor types also display MGMT deficiency [15]; by rationally combining TMZ with a DDR-inhibiting agent (TRC102), the efficacy of TMZ might be extended to malignancies beyond GBM without requiring molecular testing.

TRC102 has been developed in both intravenous (IV) and oral formulations. When combined with another IV agent, such as fludarabine [16], administering IV TRC102 does not have an impact on patient convenience. When combined with an IV agent with a longer exposure, however, repeated IV dosing of TRC102 becomes cumbersome. Instead, Gordon *et al.* combined IV pemetrexed with an oral TRC102 formulation dosed QDx4, providing a longer exposure to the effects of TRC102 [11]. Unfortunately, this oral formulation was a powder and patients were required to mix it into a liquid, making it less convenient than a capsule. While a recent study combined oral QDx5 TMZ with TRC102 delivered as a single IV infusion on day 1 of the 28-day cycles [17], single-dose designs for TRC102, whether IV or oral, produce a gap in simultaneous exposure that grows with each day of the QDx5 dosing for TMZ, as seen in the published single-dose PD data for TRC102 [17, 18]. An ongoing phase II trial of a QDx5 oral TRC102 capsule formulation and oral TMZ in patients with a first recurrence of GBM will hopefully provide additional data regarding this mode of administration; early results have reinforced the safety and tolerability of this oral combination regimen [19].

In our study, the oral capsule formulation of TRC102 combined with oral TMZ was well tolerated with a manageable side effect profile. The most common AE was anemia; one instance of grade 3 hemolysis was observed at DL8. In the expansion cohort at DL7, further grade 3 anemia events were observed, with some characterized by reactive reticulocytosis, increased bilirubin, and decreased haptoglobin in the absence of hemoglobinuria, in keeping with the extravascular hemolysis events noted preclinically. Specialty erythrocyte testing, including methemoglobin and RBC enzyme analysis, was unremarkable in all patients evaluated. These findings suggest a lack of significant underlying RBC abnormalities or predisposition to hemolysis; however, we could not identify specific molecular mechanisms by which extravascular hemolysis occurred. Importantly, all episodes of anemia were transient, reversible, treatable with transfusion, and preventable with dose reduction.

Of particular interest from any phase I combination trial including a clinically active agent such as TMZ is whether the combination seems to have greater activity than the most active of the components. The overall response rate of our trial was 7.8% (4/51) but these data cannot be directly compared with the results from prior single-agent TMZ trials due to the heterogeneity of the patient population in this trial. Instead, further assessment of the disease types that responded to the combination therapy may highlight specific tumor types with a higher likelihood of benefiting from this regimen. For example, one of two patients with NSCLC patients enrolled on the study experienced PR, contrasting strikingly with the complete lack of PRs in a phase II study of 25 patients with chemotherapy-naïve, stage IV NSCLC who received single-agent TMZ QDx5 [20]. In the case of CRC, two previous phase II trials of QDx5 single-agent TMZ in patients with pretreated, metastatic CRC reported an ORR of 12% and 10%, respectively, when utilizing prospective selection for MGMT deficiency [21, 22]. While our observation of PR in 1 out of 16 (6.3%) patients with CRC is lower than the data from these earlier studies, the discrepancy may be attributable to the fact that earlier studies selected for MGMT deficient patients who are expected to respond favorably to single-agent TMZ while our study enrolled patients irrespective of MGMT status; therefore, it would be premature to rule out possible benefit from this combination in CRC. Finally, although there is little published data regarding single-agent TMZ in patients with non-epithelial ovarian cancers, the observation that both such patients enrolled on this trial went on to experience PRs is conspicuous. Therefore, while not unambiguous evidence of greater combination than single-agent activity, we find these results worthy of additional clinical investigation.

Numerous repair mechanisms can act on the DNA adducts formed by alkylating agents such as TMZ; deregulation of these pathways is common in cancers and frequently a mechanism of drug resistance [9, 23]. To date, however, TMZ single-agent anti-cancer activity has only been directly correlated with tumor *MGMT* promoter methylation status [8]. For example, although *MGMT* methylation status was not prospectively assayed in the pivotal EORTC/NCIC trial of adjuvant TMZ in adults with glioblastoma, patients with tumors exhibiting unmethylated *MGMT* promoter regions derived less benefit [24, 25]. Promoter methylation of the *MGMT* gene leads to decreased protein expression, so other studies have evaluated MGMT protein expression directly by IHC using various thresholds to define low or negative expression [26–28]. Although *MGMT* status has not been consistently evaluated across malignancies and heterogeneity has been reported between tumors by type or organ [26, 28, 29], there is considerable literature supporting this biomarker as a predictor of response to TMZ therapy [8, 24, 26, 28–31].

Because only a subset of patients’ tumors exhibit *MGMT* promoter methylation or low MGMT expression [15], we postulated that suppressing the BER pathway with TRC102 to exploit the N-methylation of adenine and guanine might provide an MGMT-independent strategy to potentiate the efficacy of TMZ and provide clinical benefit to a wider range of patients (Supplementary Figure 1). While this study was not powered to directly address the correlation between MGMT protein levels and clinical response, the epigenetic silencing of *MGMT* in the patient who achieved a nearly complete response and the overall response rate of 4/51 (7.8%) provide some hints that the activity of this combination may not be independent of MGMT expression; however, a larger study population will be required to directly assess the role of MGMT in the efficacy of the TRC102/TMZ combination. Other molecular markers (such as extended *RAS* testing) were generally unavailable because their use was not yet widespread during the enrollment for this study. Of patients with evaluable tumor biopsies, 50% demonstrated a significant increase in Rad51 foci (with or without γH2AX and pNbs1, indicators of DNA double strand breaks), documenting that the combination produces replication stress as intended; however, these nuclear DDR biomarkers cannot distinguish between genomic injury resulting from a lack of MGMT-mediated repair or inhibition of BER.

In summary, the clinical activity of TRC102/TMZ warrants further investigation; an ongoing phase II trial of this combination in the 3 histologies where PRs were observed in the phase I study (colorectal, NSCLC, and granulosa cell ovarian) will investigate molecular characteristics that may identify the patient population benefitting from TRC102/TMZ.

## MATERIALS AND METHODS

### Patient eligibility

Patients 18 years of age or older with histologically confirmed solid tumors or lymphoma whose disease had progressed on standard therapy were eligible. An ECOG performance status ≤ 2 and adequate liver, kidney, and marrow function defined as creatinine < 1.5 x ULN, total bilirubin ≤ 1.5 × the ULN, aspartate aminotransferase and alanine aminotransferase ≤ 2.5 x ULN, an absolute neutrophil count ≥ 1,500/μL, platelets ≥ 100,000/μL, and hemoglobin ≥ 9 g/dL were required. Previous anticancer therapy, radiation, or surgery must have been completed at least 4 weeks prior to enrollment, and evidence of disease progression by staging scans was required. Patients with brain metastases were eligible if treated and stable for ≥ 4 weeks without requiring steroid and anti-seizure medications. Exclusion criteria included inability to swallow pills, uncontrolled intercurrent illness, pregnancy or breastfeeding, and gastrointestinal conditions that might predispose patients to drug intolerability and/or poor drug absorption. HIV-positive patients on combination antiretroviral therapy were ineligible because of possible PK interactions with TRC102.

### Study design

This was an open-label, single-center phase I trial in the traditional 3+3 design with an expansion cohort conducted under a National Cancer Institute-sponsored Investigational New Drug application with institutional review board approval. Protocol design and conduct followed all applicable regulations, guidances, and local policies. All protocol participants provided written informed consent (https://clinicaltrials.gov/ Identifier: NCT01851369).

Both agents were administered orally and concomitantly on days 1 to 5 of a 28-day cycle (QDx5), starting at dose level 1 (DL1; 25 mg TRC102 and 125 mg/m^2^ TMZ). DLT was defined as an adverse event at least possibly related to administration of study drugs occurring during the first cycle. A maximum of 2 dose reductions were allowed, with no re-escalation. Patients who had dose reduction without significant change in their toxicities could be reduced to 4 days of administration instead of dropping to the next lower DL. If toxicities did not resolve to retreatment criteria within 2 weeks of a new cycle, patients were taken off study. After determination of the MTD, an additional 15 patients were enrolled for evaluation of PD endpoints.

Tracon Pharmaceuticals, Inc. (San Diego, CA) and the Division of Cancer Treatment and Diagnosis, National Cancer Institute, have a Collaborative Research and Development Agreement for TRC102. Under this agreement, DCTD manufactured and supplied 25 mg capsules of TRC102 for this trial.

### Study assessments

Radiographic evaluation was performed at baseline and every two cycles to assess tumor response based on the Response Evaluation Criteria in Solid Tumors version 1.1. Adverse events were graded according to National Cancer Institute’s Common Toxicity Criteria version 4.0. At the MTD, patients with anemia underwent additional testing for extravascular hemolysis as well as erythrocyte analysis (osmotic frailty, methemoglobin, G6PD and RBC enzyme evaluation).

### Correlative studies—pharmacokinetics

Blood samples for PK analyses were collected prior to drug administration and 1, 2, 3, 4, 8, 12, and 24 hours post-dosing on cycle 1 day 1 in both the escalation and expansion cohorts, and prior to dosing on cycle 1 day 5 in the expansion phase only. All samples were centrifuged, and plasma was stored at –70°C and analyzed using published LC-MS or LC-MS/MS methods [18, 32].

### Correlative studies—pharmacodynamics

Tumor biopsies for evaluating molecular pharmacodynamic response were mandatory in the expansion phase (15 patients). Up to five tissue cores were collected at baseline and again after 4–5 days of treatment, flash-frozen in liquid nitrogen, and stored at –80°C until processing into formalin-fixed, paraffin-embedded blocks for sectioning, in compliance with clinical SOPs that preserve labile post-translational modifications like phosphorylation in core needle biopsies [33, 34]. Biopsies determined to be suitable for pharmacodynamic analysis by H&E pathology evaluation [35, 36], including adequate viable tumor content in flanking H&E sections, were analyzed with a validated, fit-for-purpose quantitative immunofluorescent assay of nuclear γH2AX, pNbs1, and Rad51 as previously published [14].

### Correlative studies—protein and gene expression profiling

Baseline MGMT protein levels were assessed by IHC, and biopsies with low tumor MGMT expression were submitted for follow-on IHC assessment of the MMR proteins MLH1, MSH2, MSH6, and PMS2; IHC was performed using a BondMax^®^ autostainer (Leica Biosystems). Optimal conditions using the autostainer included heat-induced epitope retrieval in EDTA (Leica Biosystems) for 10 mins. Primary antibody incubation (MGMT, Abcam EPR4397; MLH1, Pharmingen G168-15; MSH2, DAKO FE-11; MSH6, Abcam, EPR3945; PMS2, Abcam EPR4397) occurred at room temperature for 30 mins and was visualized using the Bond Polymer Refine Detection Kit (Leica Biosystems). Each slide was digitally imaged using an Aperio ScanScope^®^. The verification of staining performance was confirmed on a series of human xenograft cancer tissue samples. MGMT staining analyzed by an anatomic pathologist who was blinded to clinical results. Only tumor cells were evaluated (no stromal cells, inflammatory cells, blood vessels or normal background tissue) and staining intensity was compared to the background endothelial cells or hepatocytes. A tumor nuclear staining cutoff of ≥ 30% was chosen to define low MGMT expression based on results from temozolomide treatment of patients with glioblastoma multiforme [37].

The DNA methylation profile of colon carcinoma from patient 29 was generated from archival tumor tissue using bisulfite-converted DNA processed through the Infinium FFPE restoration workflow and then hybridized on a HumanMethylationEPIC BeadChip (EPIC) (Illumina, CA, USA) [38]. Array beadchips were scanned on the Illumina iScan system to produce IDAT files. To better understand the effects of *MGMT* promoter methylation on gene expression, we analyzed the colorectal cancer dataset from The Cancer Genome Atlas (TCGA) project. IDAT files from Infinium HumanMethylation450 (HM450) array, a previous generation array with most features also represented on the EPIC array, were downloaded from the NCI Genomic Data Commons (GDC) Legacy Archive (https://portal.gdc.cancer.gov/legacy-archive) [39]. TCGA RNA-seq data adjusted for batch effects used in the Pan-Cancer Atlas studies were obtained from https://gdc.cancer.gov/about-data/publications/pancanatlas.

IDAT files from HM450 and EPIC platforms were processed using the same pipeline implemented in the R package *SeSAMe* (https://github.com/zwdzwd/sesame). Specifically, the signal intensities corresponding to methylated (M) and unmethylated (U) alleles were extracted from the IDAT files by the readIDATpair function. A detection *P*-value for each probe was calculated using pOOBAH (*P*-value with Out-Of-Band probes for Array Hybridization), which is based on the empirical cumulative distribution function of the out-of-band signal from all Type-I probes [40]. The signal intensities were further processed with background correction and dye-bias correction. The background correction is based on the noob method [41]. The dye-bias is corrected using a non-linear quantile interpolation-based method using the dyeBiasCorrTypeINorm function [40]. β values, defined as SM/(SM+SU) for each locus where SM and SU represent signal intensities for methylated and unmethylated alleles, were computed using the getBetas function. β values range from zero to one, with scores of zero indicating no DNA methylation and scores of one indicating complete DNA methylation. Probes with a detection *P*-value greater than 0.05 in a given sample were masked as not available (NA). Additional experiment-independent masking of probes subject to cross-hybridization and genetic polymorphism was implemented according to the probe manifest (release 20180909) downloaded from https://zwdzwd.github.io/InfiniumAnnotation [42]. Further information on the arrays, including detailed annotation of transcription association for each probe, was obtained from the same source.

## SUPPLEMENTARY MATERIALS



## Abbreviations

AP: apurinic/apyrimidinic
APE: AP endonuclease
BER: base excision repair
CRC: colorectal cancer
DDR: DNA damage repair
DL: dose level
DLT: dose limiting toxicity
IHC: immunohistochemistry
GBM: glioblastoma multiforme
MTD: maximum tolerated dose
MGMT: O6-methylguanine DNA methyltransferase
MMR: mismatch repair
NSCLC: non-small cell lung cancer
PR: partial response
PK: pharmacokinetics
PD: molecular pharmacodynamics
RP2D: recommended phase 2 dose
RBC: red blood cell
SD: stable disease
TMZ: temozolomide
ULN: upper limit of normal
