# Supplementary material for: Phase I trial of TRC102 (methoxyamine HCl) in combination with temozolomide in patients with relapsed solid tumors and lymphomas
Source: Oncotarget. 2020 Nov 3;11(44):3959–71. doi: 10.18632/oncotarget.27784 (PMC7646836; doi:10.18632/oncotarget.27784)
Supplement: Supplementary file 1 [file oncotarget-11-3959-s001.pdf]

Phase I trial of TRC102 (methoxyamine HCl) in combination with temozolomide in patients with relapsed solid tumors and lymphomas

SUPPLEMENTARY MATERIALS

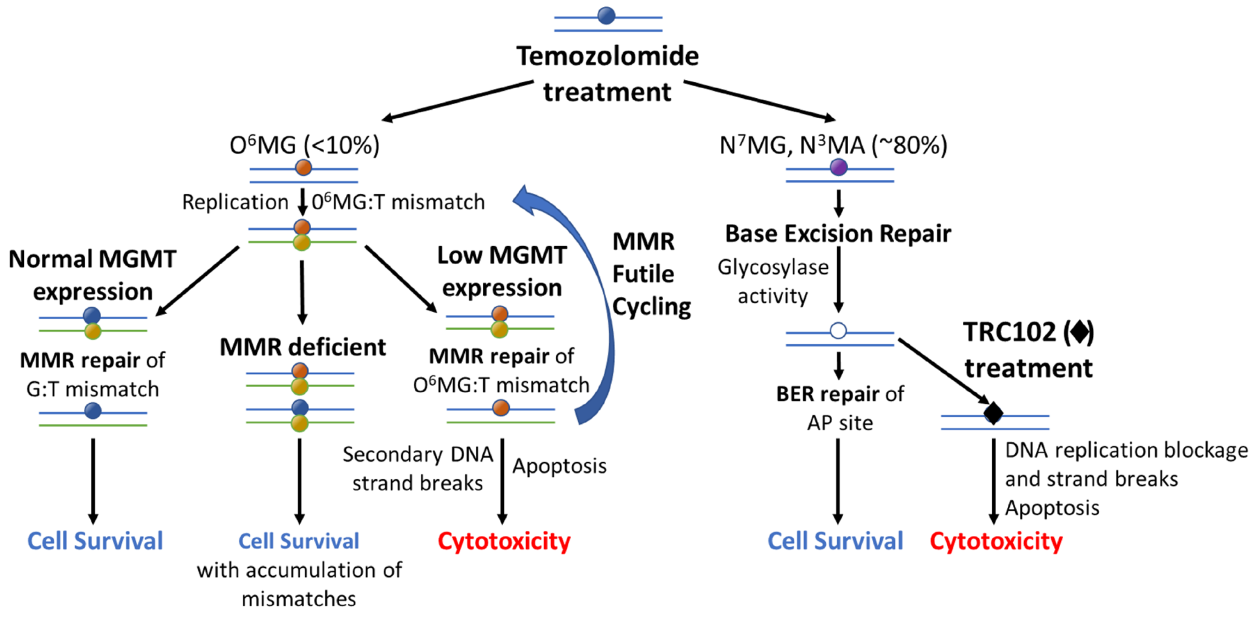

Supplementary Figure 1: DNA repair mechanisms implicated in the combination of TMZ and TRC102. Adapted from Liu & Gerson [10].
